# Knowing the entire story – a focus group study on patient experiences with chronic Lyme-associated symptoms (chronic Lyme disease)

**DOI:** 10.1186/s12875-022-01736-5

**Published:** 2022-06-02

**Authors:** M. E. Baarsma, S. A. Claassen, H. E. van der Horst, J. W. Hovius, J. M. Sanders

**Affiliations:** 1 Amsterdam UMC, location University of Amsterdam, Center for Experimental and Molecular Medicine, Meibergdreef 9, 1105 AZ Amsterdam, The Netherlands; 2 Amsterdam UMC, location University of Amsterdam, Department of Internal Medicine – Division of Infectious Diseases, Amsterdam, the Netherlands; 3 Amsterdam Infection & Immunity Institute, Amsterdam, the Netherlands; 4 Department of Language & Communication, Radboud University, Centre for Language Studies, Nijmegen, the Netherlands; 5 Amsterdam UMC, location Vrije Universiteit Amsterdam, Department of General Practice/Family Medicine, Amsterdam, the Netherlands

**Keywords:** Lyme disease, Chronic Lyme disease, Focus group, Patient perspective, Narrative analysis, Health communication

## Abstract

**Background:**

Healthcare providers frequently struggle to provide effective care to patients with chronic Lyme-associated symptoms (chronic Lyme disease, CLD), potentially causing these patients to feel misunderstood or neglected by the healthcare system. This study is the first to use a combined medical and communication science approach, and aims to assess patients’ experiences with CLD & CLD-related care, identify themes and repertories in these patients’ narrations, and provide potential ways to improve communication with them.

**Methods:**

Informed by the principles of ‘clean language’, we conducted focus groups with self-identified CLD patients (*N* = 15). We asked participants about their experiences with CLD and CLD-related healthcare. We performed thematic analyses using a bottom-up approach based in discourse analysis. We also sought to identify specific types of verbalizations (repertoires) across themes.

**Results:**

Participants thematised a heterogeneous set of CLD-associated symptoms, which they frequently labelled as ‘invisible’ to others. Their illness significantly affected their daily lives, impacting their work, social activities, relationships with loved ones, hobbies and other means of participating in society. Negative experiences with healthcare providers were near-universal, also in patients with short-lived CLD-associated symptoms. Verbalizations were notable for frequent use of communicative modes that implicitly create common ground between participants and that give a certain validity to personal experiences (impersonal ‘you’ and other forms of presupposition).

**Conclusion:**

Central themes found in CLD patients’ communication are 1. the experience of significant symptoms, 2. for which adequate relief is only rarely found from conventional medical practitioners, and 3. that are largely invisible to the outside world. Verbalizing these themes, patients use various repertoires for their shared experiences, such as a feeling of abandonment or not being heard by the medical system, feelings of loss with respect to their previous health, and the idea that they might have been better off had they been diagnosed sooner. Working with these repertoires will enable healthcare providers to establish a shared perspective with their CLD patients, thus engaging in more fruitful doctor-patient communication. We hypothesize that these findings are not unique to CLD, but may also be applicable to other conditions with an uncertain aetiology, such as Long COVID.

**Supplementary Information:**

The online version contains supplementary material available at 10.1186/s12875-022-01736-5.

## Background

Most healthcare professionals – and general practitioners in particular – will have encountered patients with chronic symptoms^1^ associated with, or attributed to an infectious disease. A prominent example is the constellation of symptoms attributed to a *Borrelia* infection, also known as chronic Lyme disease (CLD).

While frequently used by patients, the media and a small subset of medical professionals, the term ‘chronic Lyme disease’ does not refer to a specific condition with established diagnostic criteria, but rather serves as an umbrella term for various chronic LD-associated symptoms. It should be distinguished from late disseminated manifestations of LD, such as acrodermatitis chronica atrophicans or late Lyme arthritis, which are universally recognized diagnostic entities caused by an active *Borrelia* infection [1, 2]. One definition of CLD refers to persistent symptoms after treatment of confirmed LD manifestations, commonly known as post-treatment Lyme disease syndrome (PTLDS) [2, 3]. This well-known phenomenon occurs in approx. 5-10% of patients who have had LD [2, 4], but lacks a settled explanation for its pathogenesis. Most commonly, however, CLD refers to a group of patients with an array of chronic symptoms which are in some other way attributed to LD. Such CLD classifications are frequently made in the absence of any current or former LD-specific symptoms, or are not supported by conventional laboratory tests. Although research on chronic LD-associated symptoms continues, a large majority of medical professionals and biomedical researchers do not point to a persistent *Borrelia* infection as the explanation for CLD patients’ complaints, and most guidelines do not acknowledge such infections as their cause [1, 2, 5]. CLD can therefore be considered a contested illness, that is, an illness that is marked by controversy with respect to its biological origin [6].

Importantly, this does not mean that these patients are not ill. A number of qualitative studies on CLD have found that patients experience a range of symptoms with a profound impact on the ability to live their lives: from losing their job, their house or their favourite hobby, to diminished social relations and barely being able to go outside [7–10]. Many suffer not only from their symptoms, but also because of the perceived invisibility of their illness and associated lack of understanding from the people around them [8, 9].

Previous research primarily utilized individual interviews and had a distinct medical perspective [7–10]. The present study expands on this approach by focusing on the experiences that are verbalized when CLD patients of various backgrounds interact: how do they then talk about their experiences with CLD and their experiences with health providers? To investigate this, we used small focus groups that applied the principles of ‘clean language’ [11]. Focus groups are particularly suited to evoke personal disclosures about sensitive themes [12, 13], as they may arise from mutual trust and recognition among participants. Clean language in qualitative research prevents that the researchers’ professional terminology is introduced in participant interactions. It ensures that the resulting verbalizations optimally reflect the participants’ own ideas of their experiences [14]. Taken together, these techniques allow us to identify themes and concepts important to CLD patients themselves, complementing insights from (bio)medically focused research and deepening our understanding of patients’ perspective on CLD-related illness.

Such a better understanding is urgently needed [15], as physicians frequently struggle how to properly care for CLD patients and those with similar contested illnesses. Notwithstanding the possibility that a specific aetiology may be found for CLD in the future, it involves complaints that are currently categorized as –for lack of a better word– medically unexplained symptoms. Such unexplained symptoms, better known as Persistent Physical Symptoms (PPS), present a challenge for patients and medical professionals alike [16–20]. Doctors regularly experience difficulty in effective communication providing care for patients with PPS [17, 20]. These difficulties are clearly felt by patients as well, who may experience lack of empathy and understanding in their doctor [19]. This is specifically true for CLD [7, 8], and may lead to CLD patients’ sense of neglect by the conventional healthcare system [15, 21–25].

Our present approach aims to start bridging this divide. Starting point is the Language and Social Action Theory [26], which brings together different research disciplines that consider that language is an integral part of a social activity, rather than only a medium of communication [27]. In this model, patients and their context (including caregivers) apply language to not only describe, but also achieve their ‘shared realities’ [28]. Analysing talk as a social practice aims to understand how descriptions are put together and what actions they achieve. The concept of repertoires specifically helps to reveal how interaction individuals work towards common ground in their particular health context [29]. Understanding how patients communicate about their illness through the lenses of Language and Social Interaction Theory will provide healthcare providers with repertoires to discuss such aspects in relevant contexts [27]. Thus, we intend to provide an overview of themes and repertoires of patient experiences [30–32], verbalized from their own perspective, which will help medical professionals to engage in more fruitful communications with CLD patients. To this end, we formulated the following research questions:

1. Which experiences, events and interactions are significant to CLD patients with respect to their illness? Specifically, which experiences, events and interactions with healthcare professionals are significant?
2. What themes can be identified in the narratives and interactions of CLD patients on these experiences, events and interactions, and what repertoires verbalize these themes?

Thus, we explore the challenges that CLD patients face, and reflect on ways to improve communication with not only CLD patients, but also those with other post-infectious syndromes, such as post-Q fatigue syndrome or Long COVID [33–36].

## Methods

### Study design

Focus groups using the principles of clean language were organized between November 2019 and January 2020 [11]. Enrolment continued until no more new issues arose, which was after four focus groups were held. After preliminary analysis of the data generated by these four clean-language-based focus groups, we held one more focus group in August 2020 with two additional participants to check saturation [37]. In this focus group, clean language was replaced by a more directive approach to verify the validity of the preliminary results.

### Participants

Participants were recruited through purposive and snowball sampling, and were approached by either telephone or email. A total of 32 persons were approached by the researchers, of whom 11 did not respond and 6 declined to participate. The remaining 15 persons were enrolled (*n* = 13 for primary data collection, *n* = 2 for subsequent saturation check). Participants were of diverse gender and age (nine women & six men in their twenties through sixties). All participants self-identified either as CLD patient, or as someone who had had persistent LD-associated symptoms in the recent past. All were presently or formerly seeking medical treatment for their illness. Participants were recruited because they were known to be active in the Dutch Lyme community (e.g., patient organization members, Lyme internet forum contributors, etcetera), or because they had been visiting Amsterdam UMC’s Lyme outpatient clinic. As we aimed to investigate the full spectrum of patient experiences with long-lasting LD-associated symptoms, self-identification was used as inclusion criterion; potential participants were not in- or excluded based on whether their medical histories contained a confirmed LD diagnosis or positive *Borrelia* serology. We use CLD in this context to indicate all chronic symptoms attributed to LD, irrespective of whether a relationship with a *Borrelia* infection would be considered likely in medical terms.

### Study procedures & data acquisition

Potential participants were informed in advance that the goal of the study was to gain insight in the experiences of patients with long-lasting symptoms associated with LD. They were informed in advance and again on the day of the focus group (before and after the session) about study procedures, audiotaping, pseudonymisation, and the possibility to withdraw or correct data. Written informed consent was obtained prior to study inclusion. Participants were reimbursed for any travel expenses but were not paid for participation.

Focus groups were held in a non-medical conference room at either Amsterdam UMC or the Radboud University’s academic hospital. The final (i.e. saturation check) focus group was conducted via videoconferencing, as in-person meetings were impossible at that time due to COVID-19 restrictions. Focus groups were conducted by an experienced female moderator (JMS, a professor of narrative/health communication) with an assistant. The moderator was not known to the participants prior to their inclusion and had theretofore never been involved in any LD/CLD-related patient care or research. Using a semi-structured interview method, the moderator posed neutral questions to elicit participant narrative production and interaction about experiences, events and interactions, past and present, they deemed relevant with respect to their illness. This was asked with respect to patients’ social context and media context, as well as specifically with respect to their experiences, events and interactions with healthcare professionals.

Questions were posed using clean language, abstaining from statements that contained any type of judgement, and instead using only content-neutral speech to elicit, clarify, expand, or contrast participant responses [11]. More specifically, the interview method avoids evaluative content expressions (such as ‘problem’ or ‘bad’; ‘normal’ or ‘strange’), and instead invites clarifications without offering interpretations (i.e. only posing questions such as ‘what happened next?’; ‘if that happens, what do you do?’; ‘upon hearing this, how was that for you?’). This procedure allowed participants to choose their own words and freely react to others’ words, which ensured that resulting conceptualizations and evaluations were generated by participants themselves rather than partly elicited by the moderator. The topic list is provided in the Additional file 1. Focus groups lasted 60-90 minutes and had 2-4 participants each. All participants were given a summary of the findings for comments or corrections; no requests for corrections or withdrawal of data were received.

### Analysis

Audiotapes of the focus groups were transcribed verbatim by MEB or a research assistant. Data were managed and analysed using Microsoft Excel and Atlas-TI. Analyses were performed in two rounds by JMS, MEB (medical doctor specializing in LD research) and two research assistants specializing in communication and text linguistic research, among whom SAC. A discourse analysis on the transcripts of the first four focus groups was performed using a bottom-up approach based in thematic analysis for identifying communicative themes, as well as specific verbalizations (or ‘repertoires’) of these themes [30–32]. The analysts collaborated in analysing the different themes addressed by the participants. Transcripts were read repeatedly to achieve data familiarity. Theme codes were created for categories in which participants discussed experiences of symptoms, illness labelling, medical and social interactions, and related aspects. Focusing on specific types of verbalizations [38], relevant parts of participants’ talk were also labelled and grouped into repertoire categories by axial coding. After identifying a core category that indicated a distinctive theme, the transcripts were re-examined by a second coder to validate the theme’s salience by searching for additional indications and possible counter-indications. This process was repeated several times and discussed between analysts in order to arrive at an exhaustive set of relevant themes that covered the range of participants’ significant experiences when discussing CLD, and the various repertoires that verbalize these themes. Results were discussed in the fifth focus group. No additional major themes evolved, which supports the results’ validity and completeness. Exemplary cases of each theme are included as brief translated excerpts in the findings below; many additional excerpts are provided in the Additional file 1.

## Results

Results from the focus groups are presented below organized by three main themes: 1. Symptoms and their impact, 2. Situations, events and interactions regarding healthcare providers, and 3. Situations, events and interactions regarding peers. Subsequently, we analyse how these themes are verbalized in repertoires that represent shared experiences between participants. As our goal was to identify relevant themes in participants’ authentic verbalizations, we have endeavoured to keep interpretations to a minimum, focusing on the language devices that create common ground.

### Theme 1: symptoms and their impact

Participants spontaneously described having experienced 25 types of symptoms that they attribute to CLD, covering various domains and varying in their specifics per participant. Participants mentioned symptoms which could be grouped together as *fatigue* (*n* = 13/15, e.g., ‘so tired’, ‘completely exhausted’, ‘very little energy’); *cognitive problems* (*n* = 8/15, e.g., ‘you get confused’, ‘your attention span, it’s just gone’, ‘I forget everything completely’); *myalgia & arthralgia* (*n* = 7/15, e.g., ‘stiff joints’, ‘painful muscles’); *impaired motor coordination* (*n* = 5/15, e.g., ‘I’ll just fall over’, ‘walking was very tough’); *visual and/or auditory impairments* (*n* = 5/15, e.g., ‘foggy vision’, ‘so hazy’, ‘just couldn’t hear’); and *the sensation of an abnormal body temperature* (*n* = 5/15, e.g., ‘felt so feverish’, ‘[felt so] cold’, ‘flu-like complaints’).

Participants indicated the unpredictable nature of their illness, experiencing alternating periods of feeling better and feeling worse. They also described their experience that CLD manifests itself in very different and unique ways from person to person, for example with respect to symptoms, modes of treatment that work or do not work, or the course of the illness. Many participants mentioned the invisibility of their symptoms, either as inherent (i.e. that they cannot be readily seen on the outside), or resulting from efforts to make them invisible (i.e. to prevent subjecting themselves to perceived ridicule or unhelpful attitudes).

P2: And you get antibiotics or whatever treatment, and then it does something, but then after a few months, or a year, or two years, boom: there we go again.

P11: Well, then I thought: “Fine, I’ll just hide it again, and I’ll just keep going. I’ll be ‘crazy’ again.” […] No-one sees this about you, because it is like a sort of invisible handicap.

Participants discussed the impact of their symptoms in a variety of situations and contexts. They clearly differentiated between their situation before and after the onset of their illness, describing changes in their daily lives (e.g., exercise, social events, work) they were forced to make because of their illness. Participants’ symptoms and their impact evoked a variety of emotions: they described sadness and grief, but also anger.

P9: You try to keep going, to keep a job, but besides that there is little you can do. No more fun things; social contact is practically impossible. You’re too tired for everything, but you always try to keep your job and your family going.

P14: I couldn’t even alphabetically organize things [for P14’s work] because I couldn’t come up with how the alphabet goes. […] There were days where I felt completely hopeless. Everything I had worked for, I had lost. […] Right now, I’ve recovered completely. But there were days where I thought: “What if this is the rest of my life?”

### Theme 2: situations, events and interactions - healthcare providers

Participants would highlight their initial visit to a regular medical professional with respect to CLD as significant. Because of the organization of the Dutch healthcare system, patients’ quest for relief from their illness commonly starts at their general practitioner (GP), who may either diagnose and treat a patient, or may refer to specialist care in hospital. Various participants had negative experiences with their GP, with some saying they completely avoided their GP or stuck to a purely business-like relationship. However, feelings of disappointment and frustration were far from unique to general practice. Virtually all participants had some negative experience with medical practitioners.

P9: If the GP had asked something, then I would have remembered. If he had asked: “Have you been in the woods, have you had a tick bite?”, then I would have [said]: “Yeah, I did have a tick bite.” And then maybe, well, then you hope that it had been treated at that time. But that didn’t happen and he didn’t ask.

P2: You go to a doctor with the anticipation that they are going to make an effort for you, but along the way you notice that they have their own interests and ideas, and if you don’t fit into their frame of reference then you can go. Yeah, the doctor is only insulting.

Participants discussed what they missed or had wanted to see in the care from their GP or other treating physician. Though some acknowledged that GPs cannot possibly know all there is to know about every disease, many participants did note a lack of knowledge on the part of their doctor about the intricacies of Lyme disease. At the same time, they wished for a doctor who took a more holistic approach and looked beyond their own expertise. Some participants expressed regret that their doctor did not believe that certain symptoms were caused by LD, while –conversely– others expressed regret that their doctor attributed all their symptoms to LD out of a perceived ‘convenience’ to do so, rather than look further what potential other causes may underlie them.

P1: They only look at that one thing, […] but all that time no-one looked at the entire picture.

P18: What I think is a pity, is… you go through a lot in a short while, all sort of things happen, and, uhm, there’s a doctor in front of you who doesn’t want to hear it. Look, when you try to describe a symptom, you experience that for the first time, you also don’t know what the medical term is. You try to describe something, but you just notice that the other side of the table is completely disinterested.

Several participants said they had been referred to psychologists, either when their doctors could not find an organic cause to their symptoms, or concurrent with further somatic work-up. Some viewed this in a positive way, others negatively, specifically when they suspected the referral stemmed from an apparent inability to diagnose their case.

P1: Well, they can’t find anything so it must be ‘between the ears’.

Participants also mentioned seeking healthcare options outside the context of regular medical practice. They indicated that they sought out these complementary or alternative medical (CAM) practices in part because it gave them some control over their situation after being disappointed by regular medical professionals.

P11: [My strict diet] gives a feeling that you have a bit of a grip. If that’s true or not, doesn’t interest me in the slightest. It just like…

P8: That at least you’re doing something about it.

P11: Exactly, and that feels good.

P8: It gives you power.

P11: It gives you a bit of control too.

### Theme 3: situations, events and interactions - peers

While many experiences about healthcare professionals were neutral to negative in nature, their interactions with peers (e.g., friends, family, colleagues) were very varied. Some participants described feelings of not being understood or making their symptoms invisible: pretending that nothing is the matter so as to not expose themselves to insensitive or unhelpful attitudes. Others experienced great support from their peers, even remarking that other people opened up about their own personal problems after the participant told them about their CLD.

P11: In [my family’s] mind, I’m like the princess on the pea, because yeah, I always have something. I don’t have much left. I have a few good friends, who I’ve known for 30 to 50 years, who know about it, and to whom I can say “Well, I’d love to go to that birthday, for example, but I can’t make it.”

Participants also relayed their interactions with other CLD patients. These sometimes took place in person, but more often were conducted online. Some also took place within the context of the patient organization. Participants indicated that they would also read or hear about other CLD patients’ experiences. Participants appreciated the mutual support and recognition between CLD patients, while some also reported being emotionally affected or even frightened by the seriousness of others CLD patients’ condition.

P11: I saw something on TV once, some clips of someone who was in bed, completely passed out, and this and that, and I thought: ‘This isn’t me, this is not what I want to be’, so I shut it off.

P16: The [patient organization], I telephoned them. And that shocked me terribly. I called them, and I think I spoke to a very young person, and I talked about some mild complaints, not at all extremely serious, and in her second sentence she advised me to start taking antibiotics just on my own. So yeah, that shocked me a lot.

### Verbalized repertoires

As mentioned previously, we did not only look at *what* participants were saying, but also at *how* they were saying it. From this, we can hypothesize what perspective (or *framing*) is implied by these verbalizations. For these analyses, we looked at the first four (clean language) focus groups only.

Table 1 lists repertoires that participants used to verbalize their shared experiences. These expressions represent what participants would typically say when interacting about their experiences, events and interactions regarding their illness.

**Table 1:** Participants’ repertoires of CLD experiences

Summarized	Repertoire
Late diagnosis	If only someone had taken me seriously earlier and diagnosed/treated Lyme, things would not have gotten out of hand.
Abandonment	If you’re diagnosed with Lyme, you’re on your own.
Loss	I have to accept that am not half the person I used to be before chronic Lyme.
Unpredictability	It keeps coming back when you least expect it.
Lack of understanding	There are few professionals who understand me, only other chronic Lyme patients truly understand me.
Need for a holistic view	Finally, a doctor looked at the complete picture.

By using such repertoires, participants express and create common ground. They achieve this by using particular language devices that are commonly (and unconsciously) applied to represent experiences in terms that converge both individual and general aspects: *impersonal you*, *other presuppositions*, and *affirmative elaboration*. These language devices are explained and illustrated in the next subsections.

#### Impersonal you

We observed the use of various forms of phrasing that have the effect of creating a common ground between the speaker and the other persons present. One such form that we observed relatively frequently was participants’ impersonal use of generic ‘you’ (Dutch: *je*), both in describing their own experiences, and in reacting to others’ descriptions.

P14: I had exactly that. [You can explain to the doctor] just two complaints, and then you get cut off, and then it’s, uhm, okay. But then I think, I have a list of complaints

P9: Yes.

P5: Yes.

P14: You just get, just get stopped.

P9: Yeah, […] you’d think that they have to know the entire story.

The second person singular usually refers to a specific other person (canonical use: ‘You are ill’, meaning the person addressed is ill) [39], but may also take the form of a general statement implying that the experience expressed is not unique to the speaker, but is shared by others as well (impersonal use: ‘With CLD, you can’t do these things because you are ill’, meaning that all persons in such situations are ill) [39]. Such generalizations create a certain connection between speaker and addressee (‘You and I share this experience; therefore, we have something in common.’), or give a certain validity to the speaker’s experience (‘My experience is shared by others; therefore, it must be real/valid.’) [39, 40]. Impersonal *you* may simultaneously distance a speaker’s actions, opinions or experiences from themselves, enabling a speaker to talk about themselves while placing (negative) experiences at a distance (i.e. creating commonplace: ‘This happens all the time, so it is not very remarkable/cannot be attributed to me personally’) [40]. Finally, it has been argued that the impersonal second singular may evoke understanding and empathy from addressees, as these are taken along in the speaker’s narrative, in which she/he functions as that narrative’s protagonist from whom the audience is rooting [39]. In our dataset, we encountered 255 speaking turns by participants that contained an instance of impersonal use of the second person singular. This translates to 12.7% (255/2016) of all speaking turns and indicates that participants put effort in creating a connection between themselves and other attendees of the focus groups and giving a certain validity to theirs and others’ experiences. Examples are included in several of the quotations above, such as “And you get antibiotics or whatever treatment….” (P2), “You try to keep going, to keep a job, but besides that there is little you can do” (P9), or “And walking with walking poles, you get commentary…” (P3, Supplementary File) to name a few.

#### Other presuppositions

Essentially, impersonal “you” presupposes so-called common ground, but it is not the only language tool for participants to do so [41]. In general, presupposition implicitly assumes that one aspect of what is said is generally true or valid. Apart from impersonal “you”, we also observed definite description as presupposing signal [42], for instance when participants talk about ‘*that* feeling of…..’ or ‘*that* idea of…’. This implies that the speaker’s feeling or idea is not unique to them, but rather generally true and shared by others. In other words, it’s not just *a* feeling that ‘I’ have, it’s *that* common feeling that others have as well. Another example entails presenting personal experiences as generally accepted facts or certainties, which are taken for granted by the speaker and, by presupposition, also by others in the communication [41, 42].

P8: When I noticed again that I was getting ill, it did make me emotional. […]

P2: Yeah, that feeling of ‘here we go again’.

P10: And of course, the bite mark disappears.

P1: Yeah.

P10: It disappears and you never see it again.

P3: Short-term memory is really, uhm, very annoying. So, you come up with tricks for that.

Here, P10 describes her own experience with a fading tick bite mark but phrases it as to imply that this happens to everyone, and follows up this statement with an impersonal second singular. P3 similarly describes her own memory difficulties but phrases it suggesting that her problem is a problem shared by CLD patients in general.

#### Affirmative elaboration

A final observed means of creating common ground was frequent affirmation of what previous speakers have said. In a focus group, participants are neutrally invited to react on what others in the group say. A striking finding in our data is that participants –even those with divergent illness severities and durations– almost invariably actively confirmed the experiences which other participants narrated. We counted the speaking turns from the first four (exploratory) focus groups in which a participant agreed with another participant as a proportion of the total number of speaking turns by participants. While we must be careful in drawing conclusions without robust data on the ‘natural’ occurrence of agreement versus disagreement in group conversations, we think it is important to point out that as much as 256 speaking turns were found containing explicit agreement with another speaker and 369 speaking turns containing implicit agreement (e.g., expanding, nuancing or clarifying the point of another speaker). This translates to 12.7% (256/2016) and 18.3% (369/2016) of total speaking turns, respectively. However, if we only look at the 733 speaking turns that entail an actual interaction between participants and/or the moderator, we find that over 85% of those interactions are explicitly or implicitly affirmative (explicit: 256/733, 34.9%; implicit: 369/733, 50.3%). Conversely, only 3.8% (28/733) of interactions entailed an explicit disagreement with another speaker, while the remaining (80/733, 10.9%) were other types of interactions such as questions or discourse markers. These frequencies were consistent between the four exploratory focus groups. This may be explained by the tendency of participants in a conversation to take their turn at a point where they can agree and, as they generally have a preference for agreements and acceptance and to avoid disagreeing with another [43]. This type of conversational pattern is known as the collaborative achievement of agreement, aimed at keeping each other’s ‘face’ in a positive way [44].

Interestingly, though several participants believed that CLD is a very heterogeneous illness (implying that experiences are not shared), they actively created and found common ground within the heterogeneity of their experiences.

P10: You’ve had it for so long […] that you know very well….

P3: Yes

P10: …what’s the matter with you.

Mod: Okay, that…

P10: Like, I know this, this is Lyme.

P3: Yes.

P4: Yeah, exactly.

In the excerpt below, all three phenomena of common ground construction come together. In this particular fragment, participants discuss a journalistic article about suicide among CLD patients (emphasis added in bold):

P14: Long before **you** get into **the** suicidal state, there should already be help, at least psychological help.

P12: And recognition…

P14: Because I think it really comes out of desperation.

P12: **Yeah**, not being heard.

P14: **Yes**, and feeling all alone and no perspective.

## Discussion

Our study was the first to explore the experiences of CLD patients by using clean-language-based focus groups, assessing not only *what* these experiences are, but also *how* these experiences are verbalized.

### Interpreting experiences

Participants reported a heterogeneous set of symptoms, except for fatigue, which was experienced near-universally. Reported symptoms (e.g., fatigue, myalgia, cognitive problems, or sensory impairments) were largely non-specific for any particular disease, and are also frequently seen in post-infectious syndromes such as Long COVID [34], in fibromyalgia [45], in patients with PPS [46] and after treatment for cancer [47], to name a few. Similar to previous research [8, 9], many patients perceive their symptoms to be invisible, either because they are intrinsically unnoticeable for others, or because patients actively hide them for fear of being judged negatively. Unexpected fluctuations in symptoms and symptom severity were common. A considerable number of participants described the far-reaching consequences of these symptoms, which impacted their ability to work or go to school, to maintain a social life, or even to just leave the house. In these respects, CLD patients’ experiences share important similarities with those with PPS, or poorly understood conditions such as fibromyalgia or multiple chemical sensitivity [48–50].

Many participants reported negative experiences with healthcare providers and divergent experiences with people in their social environment, in line with previously reported findings on CLD [7, 8, 10, 15, 25]. Various themes could be identified in participants’ negative experiences with healthcare provides. Participants felt that their healthcare providers knew too little about LD and chronic Lyme-associated symptoms, that they do not look for a cause or diagnosis for their symptoms in a sufficient or timely manner, or that - in the absence of a confirmed diagnosis – they quickly conclude that ‘psychosomatics’ must be at play. In summary, they felt that many of the healthcare providers they had encountered were dismissive of them and their CLD diagnosis. These experiences were not unique to participants who self-identified as CLD patient, but were also shared by participants who had had persistent LD-related symptoms in the past for relatively short periods of time. Some of these negative experiences are reflected in interactions with friends, employers and the media, among others: there too, some of the patients feel that they are frequently not being taken seriously or feel judged. A subset of participants expressed that they experienced some support specifically in their contact with other CLD patients, while others remarked that learning about other CLD patients’ experiences only aggravated their distress.

### Identified repertoires

Our work adds to previous qualitative research on CLD by also looking at the communicative aspects of the focus groups. Our analyses support the notion that participants are frequently looking for a connection *with* others and validation *by* others, specifically by practitioners in the healthcare sector. This is reflected in the frequent impersonal use of ‘you’ and in the many mutual affirmations expressed by participants. This is by no means unique to CLD or contested illnesses: use of impersonal ‘you’ in a healthcare context has been observed in patient descriptions of experiences with breast cancer [51], or healthcare in general [40], and even in physician descriptions of their work [52]. In all these instances, however, the purpose was to further the notion that the speakers’ respective experiences are shared by others. This generalization is further reinforced by repertoires that our participants used to presuppose and mutually agree that there is such a thing as CLD, and that this is a genuine biological disease with a plethora of accompanying symptoms. Thus, in their conversation, participants may jointly confirm and in some respects even communicatively ‘construct’ a reality in which their own and others’ experiences merge as to substantiate the meaning of CLD. Note that this phenomenon is also not unique to CLD, but has been described to occur with respect to other medical conditions [53].

In more general terms, the interaction between a certain classification and the people being classified could be described as a variant of the looping effect [54, 55]. This term describes the process in which persons with a certain classification (e.g., CLD) are influenced in their behaviours or experiences by that classification, thus necessitating an update to the classification so that it continues to adequately describe the persons in question. A similar effect may exist in CLD patients, who collectively construct and expand the meaning of the term CLD, which may in turn influence them in the way they experience their symptoms.

This search for common ground on what CLD is and how to recover from it (or more often: learn to cope with it) does not only take place within the setting of our study. Our focus groups are merely representative of numerous interactions on internet forums, in informal discussions between patients, within the patient association, but also, for example, in the reflection and conversations on articles in popular and social media about CLD. All these interactions are significant, in the sense that they can have a formative effect on how patients view themselves and their symptoms.

### Clinical implications

We put forward the hypothesis that the CLD patients would be better served if healthcare professionals understand the repertoires in Table 1 and engage with them. This does justice to the professional role of healthcare providers as counsellors, but, more importantly, it could also prevent patients from reinforcing a collective, disabling narrative and getting ‘stuck’ therein. By taking part in the search for common ground, as described above, caregivers can start guiding the patients towards more beneficial common ground. This is not unique to CLD, as it is something that doctors frequently attempt to do with their patients. However, our findings underscore the urgency of this advice *specifically* in the field of CLD.

The aforementioned repertoires indicate that it could very well be profitable to act quickly and decisively. Early diagnosis of confirmed LD, early evidence-based treatment, early recognition of persistent symptoms − including taking these symptoms seriously and thoroughly investigating them − and providing reasoned reassurance if possible, are possible ways to do this. Healthcare providers could, for example, proactively inform patients that persistent complaints after LD are relatively common, but most often have a good prognosis and only rarely point to a persistent *Borrelia* infection [2, 4]. If patients do seek medical assistance for such symptoms, providers should always take these seriously and might consider doing a more extensive work-up, or a quicker peer-to-peer or in-person consultation than they would normally do. While this may seem excessive at first sight for non-specific symptoms which only rarely point to LD, our research allows for the hypothesis that a restrained approach in this context may in fact turn out to be intensive and expensive in the end. This hypothesis ties in with recent findings on other chronic symptoms related to an infectious disease, such as Long COVID [56], and a similar approach has also been proposed in the management of PPS [57]. In summary, we would argue that a brief but thorough (diagnostic) intervention early on has the potential to prevent patients from entrenching themselves in a disabling narrative, which is difficult to escape from.

Similarly, we would argue that self-identified CLD patients may benefit from a specific approach by healthcare professionals, not dissimilar to the way patients with other contested illnesses may be approached. It is important for the physician to realize that the CLD diagnosis may be of crucial importance in the life of the patient. While ruling out a certain disease may be nothing more than crossing off a differential diagnosis to the clinician, it may have a profound impact on the life of the patient. As Jutel [58] describes it, “[diagnosis] serves as the nexus in which the clinical encounter takes place, [it] arbitrates normality and difference, organizes a patient’s illness, and determines how resources are allocated.” Following her reasoning [16, 58], we argue that it is vital for clinicians to understand the value of a diagnosis to the patient, the absence of which deprives the patient of an understanding of their misfortunes, of an explanation to provide their friends and family for lapses in taking care of their responsibilities, and of potential avenues to a restoration of health. As one of our participants (P2) put it: “I cried out of happiness when I got the diagnosis. Finally, I know what I have.” Clinicians must therefore tread carefully when ‘taking away’ a diagnosis from a patient, specifically when not substituting it with another diagnosis of somatic origin [16]. What, then, may be the substitute that provides structure and perspective to the patient?

Mindful of the aforementioned considerations, it is understandable that CLD patients in our study indicate a desire for continued counselling by healthcare professionals to guide them towards improved health. One participant (P1) from our focus groups formulated this as follows: “I think it’s a shame, because if there were sufficient counselling, I think, you can take away something [of the symptoms and limitations caused by CLD].” Mindful of both the repertoire regarding the perceived loneliness of CLD patients and the risk of getting stuck in a disabling narrative, this counselling could very well take place through peer support groups guided by a dedicated healthcare professional. While we are not aware of any trials assessing the efficacy of such facilitated support groups, these were at least suggested for other chronic conditions [59–61].

On a final note, it seems almost superfluous to point out that an uncompassionate or even disrespectful approach by healthcare providers greatly reduces the efficacy of the care that they provide, or is even counterproductive. While we could not check the veracity of any of our participants’ experiences, it is evident that even those with relatively short-lived symptoms reported disappointing encounters with medical professionals with respect to CLD. It may also be insightful for clinicians to realize that some of the strain in the doctor-patient relationship with respect to CLD, may stem from frustration over the inability to properly diagnose a patient’s case, and that some clinicians’ out-of-hand dismissal of certain symptoms as non-somatic essentially shifts the blame of this non-diagnosis from the doctor to the patient [16]. We would argue that a more useful approach to CLD departs from the traditional dichotomy of somatic and psychological factors and rather uses the biopsychosocial model, as it more comprehensively describes the complex interplay of factors surrounding chronic conditions, including CLD [62].

### Limitations

Our study is a first effort to assess CLD patients’ experiences through focus groups and the first to include a linguistic analysis. Unfortunately, circumstances forced us to accept a minimum number of two participants for a given focus group, which limits the breadth of experiences and the possibilities for interaction between participants. We do point out that all focus groups generated homogeneous data, suggesting that experiences across all focus groups (including the one with only two participants) were similar. We must also be careful in drawing conclusions based on the relative frequency of mutual affirmation, presuppositions or informal you. With little or no research on the ‘natural’ frequency of such linguistic phenomena, it is difficult to state conclusively whether the observed frequency is significantly higher than normal. Similarly, we are also careful in attributing the looping effect that we hypothesize to occur with CLD to the contested nature of the illness. We found no other research to either confirm or reject the possibility that this also occurs in other (chronic) conditions.

## Conclusions

In summary, we find that CLD patients experience significant symptoms, for which they only rarely find adequate relief from regular medical practitioners. They explicitly and implicitly seek validation of their symptoms by healthcare providers, peers and other CLD patients. We put forward the hypothesis that CLD patients may benefit from a specific approach, including appropriate (diagnostic) interventions early in the course of the illness, as these may yield long-term benefits. Finally, it is important that medical professionals are aware of their own attitudes and behaviours towards CLD and the potential effect on the patient, so that any unhelpful approaches to this medical problem – irrespective of its precise aetiology – are avoided. This advice is quite universal to all of medicine, and will not be news to most practitioners. Yet, our findings do underscore the urgency for this approach specifically with respect to an illness as complicated as CLD. While our findings relate to CLD, we identified many similar themes in research on other diseases or contested illness. Our research therefore also provides potentially important lessons for healthcare providers who see patients with such illness, including –most prominently at this time– patients suffering from persistent complains after COVID-19.

## Supplementary Information

**Additional file 1. **Knowing the Entire Story (chronic Lyme) – BMC Primary Care – Appendix. **Appendix 1.** Topic List. **Appendix 2.** Additional Quotations. **References**.

## Data Availability

Anonymized data are available from the corresponding author upon reasonable request, subject to institutional guidelines and legal requirements.

## Abbreviations

CAM: Complementary and alternative medicine
CLD: Chronic Lyme disease
COVID(-19): Coronavirus Disease (2019)
GP: General practitioner
LD: Lyme disease
PTLDS: Post-treatment Lyme disease syndrome
PPS: Persistent physical symptoms
UMC: University medical centres

## Footnotes

1 We define a symptom as any physical sensation that is described by the patient in relation to their illness, irrespective of whether it would constitute a symptom of a certain disease in medical terms.
