# Supplementary material for: Knowing the entire story – a focus group study on patient experiences with chronic Lyme-associated symptoms (chronic Lyme disease)
Source: BMC Prim Care. 2022 Jun 2;23:139. doi: 10.1186/s12875-022-01736-5 (PMC9160505; doi:10.1186/s12875-022-01736-5)
Supplement: Supplementary file 1 — Additional file 1. Knowing the Entire Story (chronic Lyme) – BMC Primary Care – Appendix. Appendix 1. Topic List. Appendix 2. Additional Quotations. References. [file 12875_2022_1736_MOESM1_ESM.pdf]

APPENDIX WITH

Knowing the entire story – a focus group study on patient experiences with chronic Lyme-associated symptoms (chronic Lyme disease)

M.E. Baarsma, MD [1,2,3]; S.A. Claassen, MA [4]; H.E. van der Horst, MD, PhD [5];
J.W. Hovius, MD, PhD [1,2,3]; J.M. Sanders, PhD [4]

Affiliations

1. Amsterdam UMC, location University of Amsterdam, Center for Experimental and Molecular Medicine, Amsterdam, the Netherlands
2. Amsterdam UMC, location University of Amsterdam, Dept. of Internal Medicine – Division of Infectious Diseases, Amsterdam, the Netherlands
3. Amsterdam Infection & Immunity Institute, Amsterdam, the Netherlands
4. Radboud University, Centre for Language Studies, Dept. of Language & Communication, Nijmegen, the Netherlands
5. Amsterdam UMC, location Vrije Universiteit Amsterdam, Dept. of General Practice/Family Medicine, Amsterdam, the Netherlands

Contents

Appendix 1 – Topic List (Original Dutch & English translation)
Appendix 2 – Additional examples of illustrative quotations
References

APPENDIX 1 – TOPIC LIST

<u>Topic list – original Dutch</u>

- Wat gebeurde er voordat bij u Lyme werd vastgesteld?
- Hoe was dat voor u?
- Hoeveel tijd duurde het voordat bij u Lyme werd vastgesteld?
- Door wie en wanneer werd bij u Lyme vastgesteld?
- Wat gebeurde er bij u toen de diagnose gesteld werd?
- Wat gebeurde er bij uw omgeving voor de diagnose? En na de diagnose?

Contact met zorgverleners

- Hoe was de communicatie met uw zorgverlener vóór de diagnose?
- Nadat bij u Lyme werd vastgesteld, hoe was toen de communicatie met uw zorgverlener?
- Wat gebeurde er in een gesprek met uw zorgverlener?
- Wat gebeurde er bij u tijdens een gesprek met uw zorgverlener?
- Wat gebeurde er met u na afloop van zo'n gesprek? Hoe voelde u zich na zo'n gesprek?

Heden

- Wat gebeurt er momenteel in uw leven?
- Wat gebeurt er wat zorg betreft?
- Als uw zorgverlener bepaalde adviezen geeft om uw situatie te verbeteren, wat gebeurt er dan met u?
- Wat gebeurt er als u terugkijkt op de periode voor en na de diagnose?
- Wat gebeurt er met u als u denkt aan andere mensen die zich in een soortgelijke situatie bevinden?

Media

- Er verschijnen allerlei berichten in de media over Lyme, en de mogelijke gevolgen ervan en behandeling van die gevolgen. We hebben hier enkele voorbeelden. Wat vindt u van deze en andere berichten in de media over Lyme? (1-4)

Tot slot

- Zijn er nog vragen die ik niet heb gesteld maar wel had moeten stellen?

Topic list – English translation

- What happened before you were diagnosed with Lyme?
- How was that for you?
- How much time did it take before you were diagnosed with Lyme?
- By whom were you diagnosed with Lyme, and when was this?
- What happened to you when you were diagnosed?
- What happened to the people around you before the diagnosis? And after?

Contact with healthcare providers

- How were your interactions with your healthcare provider before the diagnosis?
- How was your communication with your healthcare provider after you were diagnosed with Lyme?
- What happened during the conversation with your healthcare provider?
- What happened to you during the conversation with your healthcare provider?
- What happened to you after such a conversation? How did you feel after such a conversation?

The present

- What is currently happening in your life?
- What is happening in terms of healthcare?
- If your healthcare provider gives you advice on how to improve your situation, how do you experience that?
- How do you look back on the period before and after the diagnosis?
- What happens to you when you think of other people in a similar situation?

The media

- There are all sorts of stories in the media on Lyme, the possible consequences thereof, and treating those consequences. We have several examples here. What is your opinion of these and other news stories on Lyme? (1-4)

In conclusion

- Are there any questions I should have asked, but did not?

APPENDIX 2 – ADDITIONAL QUOTES

The section below lists additional examples of illustrative quotations, sorted by theme and subtheme. It is not an exhaustive list.

Symptoms and their impact

Invisibility

P2: I've had to give up so much, and no-one sees. That's impossible to comprehend. [..] People hide things, because otherwise no-one will take them seriously.

P3: And walking with walking poles, you get commentary [from people]. Like, you're walking somewhere and then 'Yodelayheehoo', as if I…. Well, then I really shouldn't respond. I can walk with these poles, they give me freedom. But because it isn't visible… You could better have a broken leg, people will see that.

P11: Well, then I thought: "Fine, I'll just hide it again, and I'll just keep going. I'll be 'crazy' again." […] No-one sees this about you, because it is like a sort of invisible handicap.

Heterogeneous experiences

P1: You [P10] experience a lot of benefit from homeopathy, but that is something that wouldn't work for me at all, because you experience such a different effect of Lyme.

P3: [Doctors] cannot properly explain or differentiate complaints, because –as you [P1] say– there is some much variation.

P18: Yeah, I recognize that a lot. That it's really varying, and that you have relapses unexpectedly.

Impact of symptoms

P8: I used to run marathons before that time. […] I exercised a lot, I loved working out. […] Well, not much is left of that. Well, so, I hate it. Uhm, I've cancelled four or five appointments in the past two weeks.

P11, after becoming emotional: It has had such an impact on my life. On the decisions I did or did not make, or was forced to make.

P18: The first three months, when I really, well, was out of it, […] there were, without reason, a lot of really intense of emotions, uhm, anger, uhm, or sadness too.

Situations, events and interactions - healthcare providers

General practitioners (GPs)

P1: Then we went back to the GP, like: “Could it have been [Lyme], because I’ve had that ring” and nothing was done with that and now we’re so many years on.

P2: From the first moment on, I’ve felt left in the cold.

P12: Yeah, the not-being-taken-seriously-by-the-GP, that’s something I can only confirm.

Other healthcare providers

P4: I’ve been operated on my eye and then [my Lyme doctor) asked the ophthalmologist: ‘Well, take some fluid from the eye to check if it’s in the eye, the Lyme disease.’ But he wouldn’t. He thought it was… well… [But the eye] is open, you can reach it. […] I was so mad at that man.

P4: They don’t say it, but [their advice is] ‘learn to live with it’.
P10: Yeah, and I don’t agree with that. That way, certainly as a doctor, you pass on your responsibility, because you don’t continue looking.

P11: That paternalistic attitude of those doctors. That I should toughen up, while I couldn’t do anything anymore. […] Doctors have so much power, they don’t only decide the definition of health, but they also decide our income, and uhm, well, socially. But they have sort of a god-like status.

P14: After a lot of pain and effort, I was referred to a neurologist, and my first conversation with him went like: “Hi, I’m Mrs. P14”. And his response was: “Aaah, P14, I’m sceptical [about the suspicion of neuroborreliosis].” He didn’t even say his name.

What healthcare providers should have done

P3: You’re eyes, you’re a heart, you’re a neurological system, but there is not a single doctor that looks at you as a whole. […] My biggest problem is that you get chopped up into bits.

P8: Well, I understand that GPs cannot know every disease, that’s perfectly normal to me. But that they have a protocol, with ‘this is it, it’s yes or no’. […] The idea that you should keep remain doubtful as a doctor, that’s gone.

P14: My GP at one point asked: “Have you looked on Google?” And I said: “Yes”, and then he said: “Well, I think it’s good that you continue, because I don’t know anything about it myself.” I think it would make a big difference if a GP […], when a patient comes to talk about a blood test for Lyme, that they read up about it.

The relation between soma and psyche

P2: They are not willing to think further about what it *could* be. Because you immediately get referrals to all sorts of other departments. The psychiatry department, to uhm, psychologists.

P3: The only one I haven't visited is a psychiatrist, as in I wasn't imagining it. But I do hear that from other Lyme patients, that they say that to them.

P6: I visited so many hospitals and specialists, and each time I was sent away. One time, I, uhm, I even heard, like: "I want to give you a referral to a psychiatrist." […] I am not crazy, I know what is wrong with me.

P14: [My occupational health doctor] said: "I want you to go to a psychologist immediately", in case it was a burn-out. […] That did give me some support.

Complementary and alternative medicine

P2: I have an orthomolecular doctor, who treats me to this day.

P2: The uncertainty you get into, and the complaints that appear, that you get nightmares, that you don't sleep, that you get confused. That was very tough, and if you're without any help, then, what do you do? So yeah, at a certain point I fled to well, uhm, a complementary medical practitioner.

P4: I just try to do something. […] You can just go sit on the couch, but that's not gonna help you either.

P6: I've been treated very differently there [in a private clinic abroad] than in the Netherlands.
Mod: And what feeling did that give you?
P6: Well, certainly the feeling that you're taken seriously. […] They did very extensive testing, for days. A lot of blood tests were done and something came out of that and the treatment plan that they wrote for me, it worked.

P5: I go to Thailand each year to buy medication.
P9: Are you still on antibiotics then?
P5: Yeah, yeah, it's antibiotics together with [hydroxychloroquine] and [an anti-fungal drug]. […] I take them continuously.

P6: [Treatments in private clinics abroad] cost me about 25 000 euros, and if you don't have that, then you're at the mercy of the gods in the Netherlands.

P9: I've been to Germany two years ago now, and I'm convinced that it's one of the best therapies for chronic Lyme, it's a hypertonic treatment.

Situations, events and interactions - peers

Friends and family

P2: I've lost so many friends, because, well, I can't keep up with them anymore. […] My family, they're all doctors and nurses and that kind of thing. And well, they think it's all a weird story and they think I should just conform to their norms, so to speak. Yeah, 'you shouldn't whine'.

P14: My colleagues came over to hoover, to cook, do groceries, walk the dog. […] That was heart-warming.

P18: I've spoken to some people, and the moment that you're walking around with this, there are so many people around you who pop up having experienced the same thing.

P1: People close to me know. [I can say:] "Yeah, I know we had plans, but sorry I can't make it." [And they will be like:] "Yeah, it's fine, we'll do it some other time. Get well soon."

P8: Going to concerts, I can't do. Dinner with people, having fun with friends, I can't do. All those things you do in the evening, forget it. […] Other people understand, that's not the issue, but I do think: does it ever stop? […] I never had the feeling that the people around me didn't take me seriously.

P10: I have no-one. The last four, four-and-half, five years, none of my friends have come over to my house. I always had to go someplace. And they're busy, and that's not strange and I don't mind, but no-one ever comes to my place.

Other CLD patients and the patient association

P2: It just gets to you, like, if you hear from yet another one [who killed themselves], then it comes really quite close.

P4: You see a lot on the internet, you hear from other patients: 'I've been there, you know, I've done the alternative medical thing, I've done ozone therapy too', you know. […] It didn't help shit.

P14: Because of many of these types of articles, I've doubted myself so often, like, is it psychological after all?

P15, reading an article entitled 'Will [name] ever get rid of her chronic Lyme?': Yeah, that's tough. […] It's much worse than what I thought it could be.

P16: [As] soon as you get anywhere near this disease, certainly online, well, it's a can of worms you're opening, with –with, primarily a lot of anger, is what I've noticed.

P1: I am a busy bee. I like doing things. […] And you know, because I have been able to do so much with the [patient organization] and was able to speak to clients.

P6: Well, I sometimes go to meetings of the [patient organization], and you see, and also speak with a lot of people, you see people in a wheelchair and then I'm like, 'well, I'm happy I am still able to walk'.

P18: It's ridiculous, scaring so many people, exactly what P16 is talking about. You just get so much anxiety. […] That there are a number of people that get really ill, does not mean that everybody will get ill.

P18: Through an internet forum, uhm, I met several people, who I later also encountered in real life. […] What we did was look up a lot of literature and discuss that. See if we could do something with it ourselves. There were several people who were very critical in this respect, people who had gone to university.

Types of verbalizations

Impersonal 'you'

P1: Because that was suggested [that P1 was 'faking it' during physiotherapy], you quit, and then it stops for you there and you start looking elsewhere.

P4: For the people around you, it's really difficult, because you can hardly see it, but you feel so miserably.

P6: So you go into the medical circuit and then you get one negative test result after the other.

P6: Because you get handicapped more and more. Walking is more difficult, […] lifting up a cup of tea or whatever. It's all getting more difficult. […] Your body… you get more and more physical limitations.

P8: Yeah, well, you know, you just want someone to do something, that's it.

P10: What do I do when I go see a physician [for my complaints] and then there's nothing, nothing to see, nothing the matter, I feel nothing. And then you go home, and there you feel the complaint again and that's something you cannot control.

P11: You can't do anything yourself, you're side-lined.

P14: It's not just regular flu, it's so much worse. […] You really can't do anything anymore.

P14: You have zero energy for doing something else, except, you know, make lunch.

Affirmative elaboration

P3: Yeah, the hazy vision, what you [P4] said. You also said something like complaints that come in waves. I also still have that.

P14: The most recognizable, what you're saying too, is that it's so different, it's not just regular fatigue.
P5: Yes.
P14: It's not just regular flu, it's so much worse.

Mod: So what you encounter is so…
P9: It's so diverse.
P5: Yes.

REFERENCES

1. Aantal Lyme-patiënten in 20 jaar verviervoudigd: NOS; [updated 17 April 2018. Accessed 18 February 2022]. Available from: https://nos.nl/artikel/2227559-aantal-lyme-patienten-in-20-jaar-verviervoudigd.
2. Komt Berdien ooit nog van haar chronische Lyme af? : NOS; [updated 6 April 2017. Accessed on 18 February 2022]. Available from: https://nos.nl/op3/artikel/2166811-komt-berdien-ooit-nog-van-haar-chronische-lyme-af.
3. 3 op 10 lymepatiënten denken aan zelfmoord: RTL Nieuws; [updated 19 April 2019. Accessed on 18 February 2022]. Available from: https://www.rtlnieuws.nl/lifestyle/gezondheid/artikel/4676716/lyme-zelfmoord-tekenbeet-teken-ziekte.
4. Leven met lyme - of is het een andere ziekte? : NRC Handelsblad; 2018 [updated 6 November 2018. Accessed on 18 February 2022]. Available from: https://www.nrc.nl/nieuws/2018/11/06/leven-met-lyme-of-is-het-een-andere-ziekte-a2754180.
